# Left Cervical Approach for Unrecognized Proximal Tracheoesophageal Fistula in an Adolescent following Neonatal Esophageal Atresia Repair: A Case Report

**DOI:** 10.70352/scrj.cr.26-0137

**Published:** 2026-07-01

**Authors:** Kazuhisa Fukuura, Maho Inoue, Aoi Matsuzaki, Hiroki Iwai, Kazuya Mimura, Masafumi Iguchi, Shohei Takayama, Kiyokazu Kim, Shigehisa Fumino, George Iwata, Shigeru Ono

**Affiliations:** 1 Department of Pediatric Surgery, Kyoto Prefectural University of Medicine, Kyoto, Kyoto, Japan; 2 Department of Pediatric Surgery, Kyoto Chubu Medical Center, Nantan, Kyoto, Japan

**Keywords:** esophageal atresia, tracheoesophageal fistula, left cervical approach, adolescent, missed fistula, bronchoscopy

## Abstract

**INTRODUCTION:**

Esophageal atresia with both proximal and distal tracheoesophageal fistulas (TEF) is rare, and a proximal TEF may be overlooked during the initial neonatal surgery. However, reports describing the surgical management of residual proximal TEF beyond infancy are limited. Herein, we report a case of a residual proximal TEF that was unrecognized during neonatal repair of esophageal atresia, remained undiagnosed until adolescence, and was successfully resected using a left cervical approach.

**CASE PRESENTATION:**

A 14-year-old boy with recurrent aspiration pneumonia was diagnosed with a residual proximal TEF and referred to our department for further management. He had a history of primary esophageal anastomosis for esophageal atresia during the neonatal period and was diagnosed with Gross type C esophageal atresia based on intraoperative bronchoscopy. He subsequently experienced recurrent aspiration pneumonia for many years. At 14 years of age, videofluoroscopic swallowing study, chest CT, and esophagogastroduodenoscopy revealed a residual proximal TEF at the level of the third thoracic vertebra (Th3). Under endoscopic guidance, a guidewire was introduced through the esophagus into the fistula and positioned within the trachea in a U-shaped configuration. Intraoperative fluoroscopy demonstrated that the fistula location corresponded to the left supraclavicular cervical region, and surgery via a left cervical approach was done. The guidewire was used to localize the proximal TEF intraoperatively by palpation and fluoroscopic guidance, and the fistula was successfully resected.

**CONCLUSIONS:**

Even in adolescent patients with a larger body habitus, a guidewire-assisted left cervical approach is an effective surgical option for residual proximal TEF located at the Th3 level.

## Abbreviations

EGD
: esophagogastroduodenoscopy
EGJ
: esophagogastric junction
RLN
: recurrent laryngeal nerve
TEF
: tracheoesophageal fistula
Th
: thoracic vertebra

## INTRODUCTION

TEF associated with esophageal atresia arises from abnormal separation of the trachea and esophagus during embryonic development. Most patients with esophageal atresia presented with Gross type C, in which a single distal TEF was noted, and usually undergo esophageal anastomosis with distal TEF resection. Persistent or recurrent respiratory symptoms after surgery raise concern for a recurrent TEF, which is a well-known postoperative complication due to anastomotic leakage.

On the other hand, a “missed fistula,” defined as a proximal TEF overlooked during the initial surgery, is rare but represents an important pitfall, particularly in cases of esophageal atresia with TEF in both the proximal and distal esophagus. Missed fistulas are generally managed surgically using treatment strategies similar to those for H-type fistula, which is characterized by a congenital TEF without esophageal discontinuity and is usually diagnosed later in life.^1)^

Herein, we report a case of residual proximal TEF that remained undiagnosed for a prolonged period after esophageal atresia repair. The fistula was successfully resected via a left cervical approach, with precise intraoperative localization achieved using guidewire-assisted fluoroscopy.

## CASE PRESENTATION

A 14-year-old boy presented to the emergency department of a referring hospital with aspiration pneumonia.

His perinatal history was notable for esophageal atresia diagnosed shortly after birth at another institution. Definitive repair was performed on day 11 of life. Intraoperative bronchoscopy identified a single distal TEF located 1–1.5 cm proximal to the tracheal bifurcation, and Gross type C esophageal atresia was diagnosed. Through a right thoracotomy, the fistula between the distal esophagus and the trachea was identified and divided, followed by primary esophageal anastomosis. The immediate postoperative course was uneventful, and he was discharged on POD 37. At approximately 11 months of age, he developed inspiratory stridor and coughing during feeding. Between the ages of 5 and 13 years, repeated evaluations—including laryngoscopy and upper gastrointestinal contrast studies—were performed; however, no definitive diagnosis was established. From the age of 7 years, he was followed by a pediatrician at the referring hospital for short stature. Around the same period, recurrent vomiting and aspiration pneumonia developed, resulting in frequent emergency department visits.

During the present admission, a comprehensive re-evaluation was undertaken in collaboration with Department of Pediatric Surgery in Kyoto Prefectural University of Medicine (hereafter, our institution). EGD revealed a fistulous opening 16 cm from the incisors (**Fig. 1**). Advancement of the endoscope through the fistula demonstrated mucosa with a protrusion suspicious for tracheal cartilage, raising suspicion of TEF. A videofluoroscopic swallowing study showed contrast material entering the trachea (**Fig. 2**). Chest CT performed immediately after the swallowing study confirmed the presence of contrast material within the trachea. The fistula was located at the level of the Th3, with the esophageal orifice situated cranial to the tracheal orifice (**Fig. 3**). During a second EGD, a guidewire was successfully advanced from the esophageal orifice through the fistula into the trachea under concomitant bronchoscopic visualization (**Fig. 4**).

**Fig. 1:**
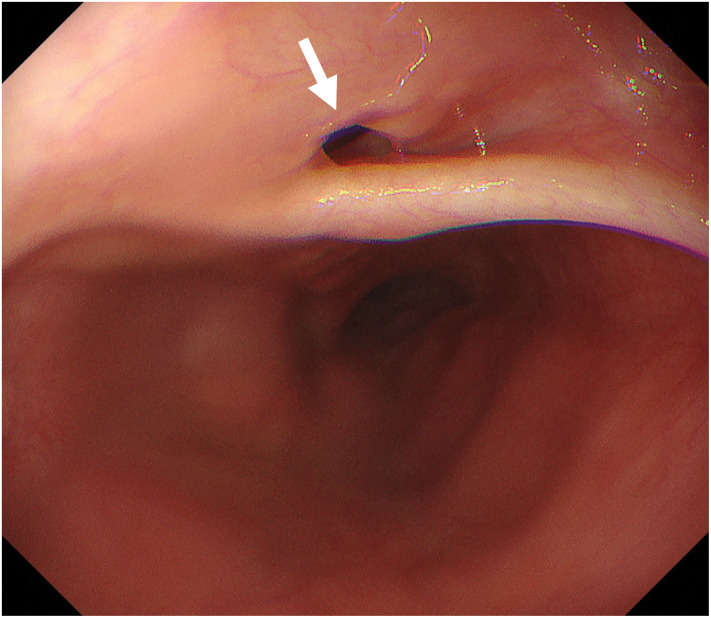
Upper gastrointestinal endoscopy showing a fistula (white arrow). The fistula was identified on the ventral side of the esophagus at 16 cm from the incisors.

**Fig. 2:**
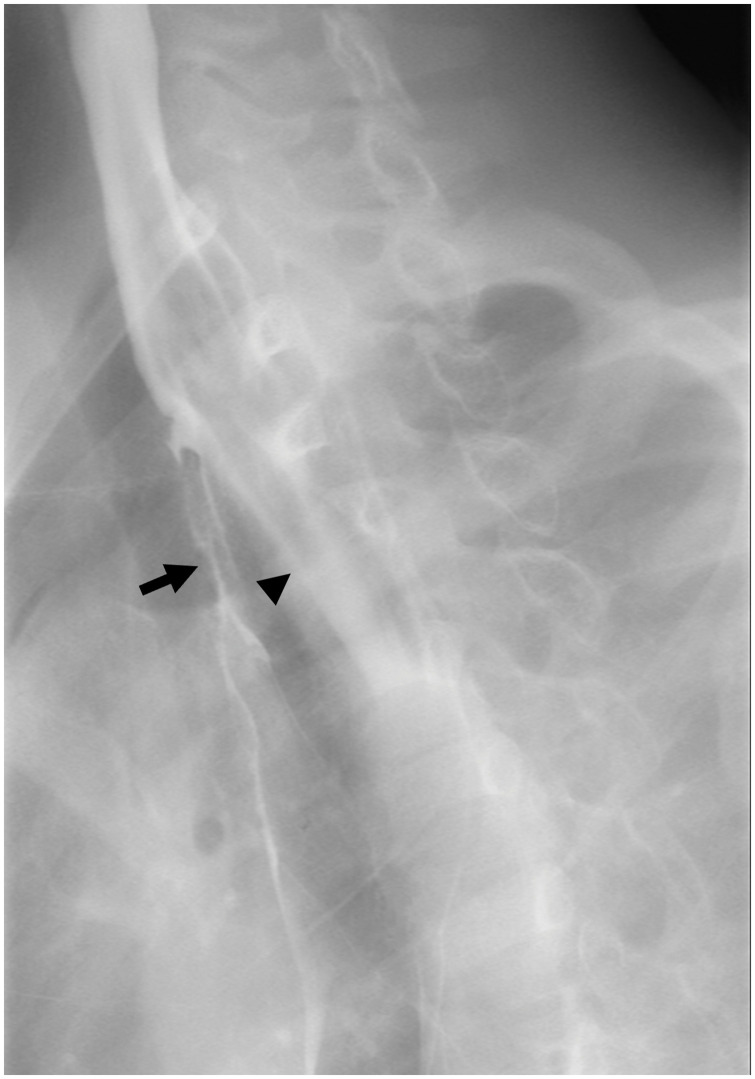
Videofluoroscopic swallowing study. Contrast material is seen entering the trachea (black arrow). The esophagus is indicated by the black arrowhead.

**Fig. 3:**
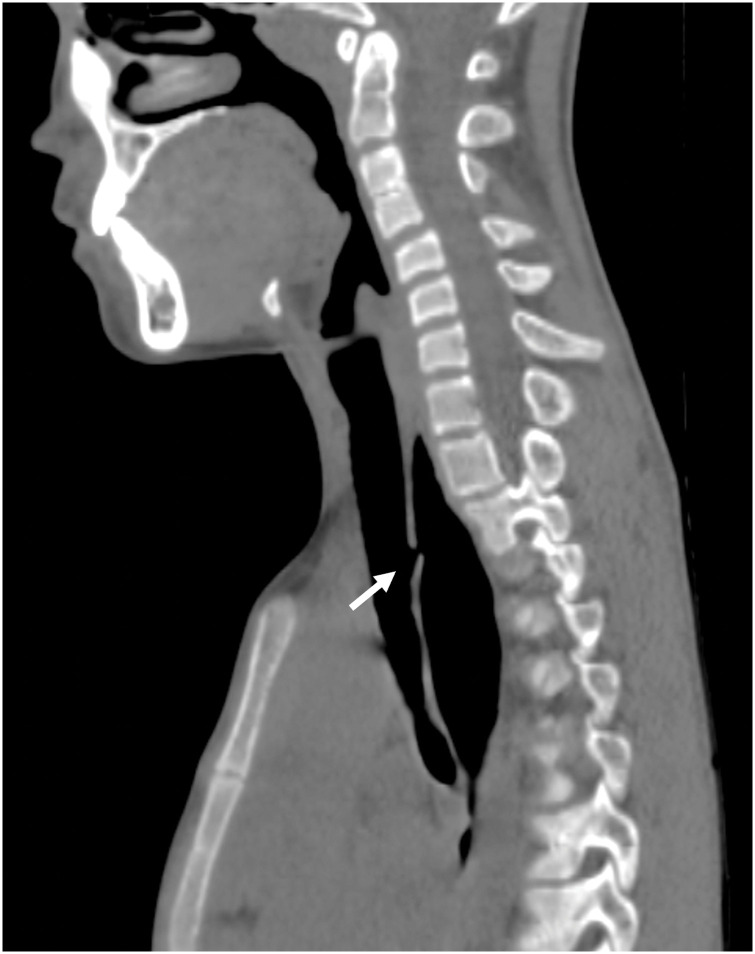
Neck and chest CT image (sagittal view). The CT demonstrates that the esophageal orifice (white arrow) of the fistula was located at a higher level than the tracheal orifice.

**Fig. 4:**
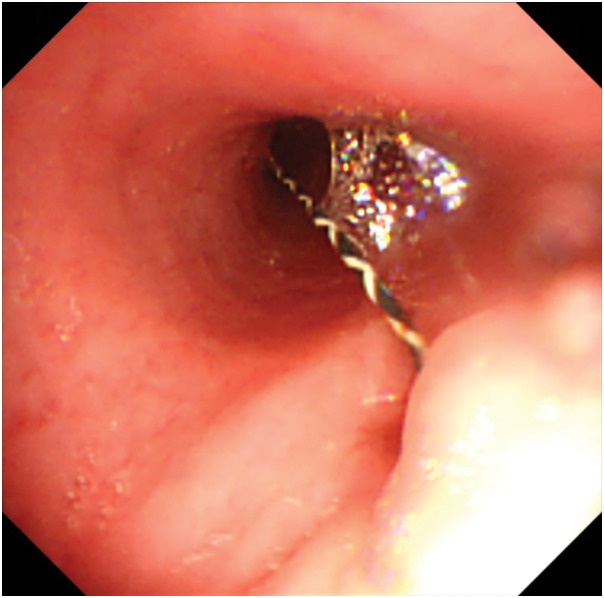
Bronchoscopic finding. The bronchoscopic view obtained after inserting a guidewire into the fistula via EGD, demonstrating the guidewire within the trachea. EGD, esophagogastroduodenoscopy

Based on these findings, the patient was diagnosed with a residual proximal TEF that had been overlooked at the time of neonatal repair. He was referred to our institution for surgical management. On admission, his body weight was 32.6 kg (−2.0 standard deviation (SD)) and his height was 151.4 cm (−1.5 SD).

Under general anesthesia, a 0.025-inch, 300-cm angled guidewire was advanced from the esophageal side into the trachea through the proximal TEF under EGD guidance. The guidewire was then grasped on the tracheal side using rigid bronchoscopy, retrieved through the oral cavity, and configured in a U-shape. Fluoroscopy demonstrated that the apex of the U-shaped loop, corresponding to the fistula, was located in the left anterior cervical region just above the clavicle on the body surface (**Fig. 5**). A 4.5-cm left cervical skin incision was made from just above the suprasternal notch extending toward the left supraclavicular region. The sternocleidomastoid muscle and internal jugular vein were retracted laterally by using a nerve hook. Weitlaner retractors were used to stabilize the operative exposure. The esophagus was dissected circumferentially, encircled with a vessel loop, and gently retracted to facilitate dissection in the plane between the tracheal and esophageal walls. The RLN and the thoracic duct were not identified within the operative field. After careful dissection along the tracheal wall, the TEF was identified with the aid of guidewire traction and fluoroscopic confirmation (**Fig. 6**). The fistula was circumferentially dissected and divided. The esophageal and tracheal wall defects were closed longitudinally with 7 interrupted 4-0 polydioxanone sutures (Ethicon, Somerville, NJ, USA) each.

**Fig. 5:**
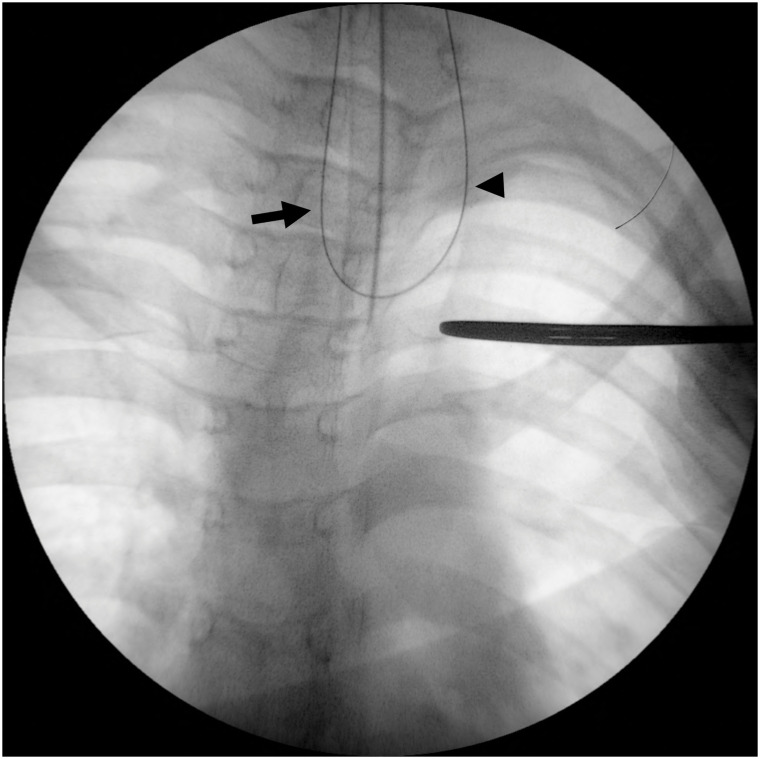
Intraoperative fluoroscopic image. Fluoroscopy demonstrated that the apex of the U-shaped guidewire corresponding to the fistula was located at the left anterior cervical region just above the clavicle on the body surface. The black arrow indicates the tracheal side, and the black arrowhead indicates the esophageal side.

**Fig. 6:**
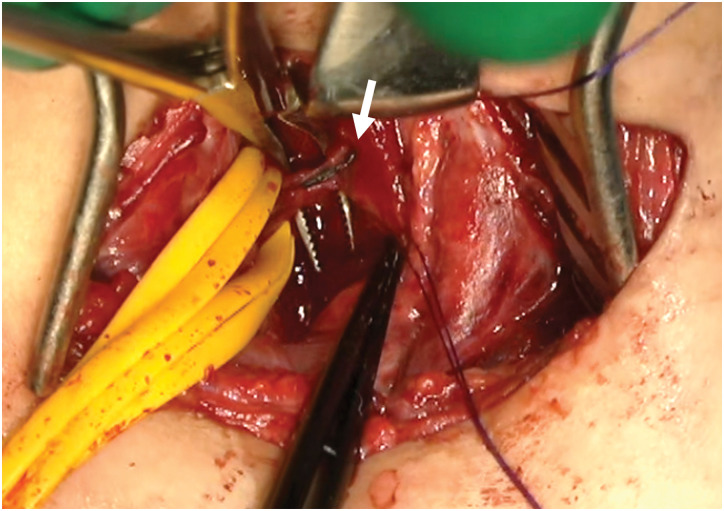
Intraoperative finding. The fistula was precisely localized using a guidewire (white arrow) traction and fluoroscopy. Left, tracheal; right, esophageal. Upside, cranial; downside, caudal.

The postoperative course was uneventful except for transient RLN palsy. Oral intake was resumed on POD 3, and the patient was discharged on POD 7. At 4 months of follow-up, there was no evidence of recurrence.

## DISCUSSION

The Gross classification is widely used to categorize esophageal atresia; among its subtypes, Gross type D esophageal atresia, characterized by the presence of both proximal and distal TEFs, is considered rare. A nationwide Italian survey conducted in 2012 reported an incidence of esophageal atresia of approximately 3.33 per 10000 live births. According to subtype distribution, type C accounted for 89%, type A for 5%, type E (H-type) for 3%, type B for 2%, and type D for 0.7%.^2)^ However, numerous case reports have discussed the difficulty of identifying proximal fistulas,^3,4)^ suggesting that many cases of Gross type D esophageal atresia may initially be misdiagnosed as type C. In a single-center case series from a pediatric hospital in China, 14 of 386 patients (3.6%) were diagnosed with Gross type D esophageal atresia. Of these, 9 cases (64.3%) were diagnosed preoperatively or intraoperatively, whereas the remaining 5 cases were identified postoperatively due to detection of a proximal fistula.^5)^ Recent reports have shown a higher incidence of proximal fistulas (4.9%–5.7%),^6,7)^ which has been discussed as being attributable to inadequate bronchoscopic examination.^6)^ Regarding bronchoscopic findings, Summerour et al. reported 4 characteristic features of proximal fistulas compared with distal fistulas: location just below the vocal cords, extremely small fistula size often appearing as a shallow depression, lack of opening during positive-pressure ventilation, and the presence of air bubbles from the fistula following esophageal insufflation.^8)^ Given these anatomical characteristics, proximal fistulas are prone to being overlooked, and careful bronchoscopic examination is essential. In the present case, several findings supported the interpretation that the lesion represented a missed proximal fistula rather than a recurrent TEF.

Residual proximal fistulas are reported to be commonly located at the level of the Th1 or Th2, and a cervical approach is therefore required in many cases.^9)^ However, thoracoscopic repair for Gross type D esophageal atresia has also been reported,^10)^ indicating that the surgical approach should be selected according to fistula level. Because residual proximal fistulas are anatomically and clinically similar to H-type TEF, we based our surgical approach on treatment strategies used for H-type esophageal atresia. A systematic review of pediatric H-type esophageal atresia cases reported that a cervical approach was commonly selected when the fistula was located above the Th2 level, whereas thoracoscopic or open thoracic approaches were considered when the fistula was located below Th2.^11)^ However, another systematic review reported variability in the proposed anatomical threshold, with some studies using the Th3 level, the first rib, or the tracheal bifurcation as reference points.^1)^ No definitive consensus has therefore been established. Moreover, the majority of reported cases are in infants, making the choice of approach for a Th3-level fistula in an adolescent patient particularly challenging. Placement of a guidewire within the proximal TEF and confirmation under fluoroscopy facilitated accurate intraoperative localization and assisted in determining whether the fistula was accessible via a cervical approach before skin incision.

Regarding laterality, a systematic review comparing right- and left-sided cervical approaches reported that a right cervical approach was used in 76.9% of cases and a left cervical approach in 13.2%, with no significant differences in complication or mortality rates.^1)^ Although the right cervical approach is generally favored because of anatomical advantages such as reduced risk of thoracic duct injury and RLN palsy,^12)^ several reports have described successful outcomes following a left cervical approach.^12–14)^ In the present case, intraoperative fluoroscopy demonstrated that the fistula location corresponded to the left supraclavicular cervical region, and a left cervical approach was therefore selected. The procedure was completed without major complications, and the postoperative course was favorable, suggesting that a left cervical approach can be a viable option depending on fistula location.

The use of a guidewire for intraoperative localization during cervical resection of a TEF was first described in detail by Garcia et al.^15)^ Although their study focused on recurrent fistulas, subsequent reports demonstrated that guidewire placement was associated with shorter operative times and hospital stays.^16)^ Therefore, this technique is considered to be highly useful for intraoperative identification of TEF.

In the present case, the RLN and thoracic duct were not identified intraoperatively; however, several strategies have been reported to reduce the risk of injury. Intraoperative identification of the RLN remains the gold standard,^17)^ and meta-analyses suggest that intraoperative neuromonitoring can significantly decrease the risk of nerve injury.^18)^ Maintaining a clear operative field with minimal bleeding and avoiding the use of energy devices near the nerve are also important.^19)^ For the thoracic duct, accurate anatomical recognition is also essential, and indocyanine green fluorescence imaging may help its identification.^20)^ In cases where the thoracic duct is exposed during surgery, performing a Valsalva maneuver may facilitate intraoperative detection of chylous leakage.^21)^

## CONCLUSIONS

We reported a case of the esophageal atresia with a residual proximal TEF that had been unrecognized at the time of neonatal repair. Proximal fistulas are prone to being overlooked, and careful bronchoscopic examination is therefore essential. This case demonstrated that a left cervical approach with guidewire-assisted intraoperative fluoroscopy allowed accurate localization and favorable results in the outcome.

## DECLARATIONS

### Availability of data and materials

The datasets supporting the conclusions of this article are included within the article.

## References

[1] Parolini F, Morandi A, Macchini F, et al. Cervical/thoracotomic/thoracoscopic approaches for H-type congenital tracheoesophageal fistula: a systematic review. Int J Pediatr Otorhinolaryngol 2014; 78: 985–9. 24856837 10.1016/j.ijporl.2014.04.011

[2] Pini Prato A, Carlucci M, Bagolan P, et al. A cross-sectional nationwide survey on esophageal atresia and tracheoesophageal fistula. J Pediatr Surg 2015; 50: 1441–56. 25783403 10.1016/j.jpedsurg.2015.01.004

[3] Afzal M, Attal MB, Tabash AA, et al. Esophageal atresia with proximal and distal trachea-esophageal fistulae. J Pediatr Surg Case Rep 2022; 76: 102106.

[4] Menso JE, Reijntjes MA, Oomen MW, et al. Missed proximal tracheoesophageal fistula (TEF) in a neonate with type D esophageal atresia. European J Pediatr Surg Rep 2024; 12: e4–6. 10.1055/a-2227-6389 PMC10781516 38214009

[5] Wang D, Zhao Y, Zhang Y, et al. Diagnose and treatment for type D congenital esophageal atresia with tracheoesophageal fistula. Pediatr Investig 2024; 8: 37–43. 10.1002/ped4.12410 PMC10951482 38516136

[6] Bax KN, Roskott AM, van der Zee DC. Esophageal atresia without distal tracheoesophageal fistula: high incidence of proximal fistula. J Pediatr Surg 2008; 43: 522–5. 18358293 10.1016/j.jpedsurg.2007.10.034

[7] Parolini F, Morandi A, Macchini F, et al. Esophageal atresia with proximal tracheoesophageal fistula: a missed diagnosis. J Pediatr Surg 2013; 48: E13–7. 10.1016/j.jpedsurg.2013.04.018 23845651

[8] Summerour V, Stevens PS, Lander AD, et al. Characterization of the upper pouch tracheo–esophageal fistula in oesophageal atresia. J Pediatr Surg 2017; 52: 231–4. 27939205 10.1016/j.jpedsurg.2016.11.009

[9] Boybeyi-Turer O, Soyer T. Tracheoesophageal fistula after esophageal atresia repair: recurrent, missed or acquired. Curr Chall Thorac Surg 2022; 4: 26.

[10] Rice-Townsend S, Ramamoorthy C, Dutta S. Thoracoscopic repair of a type D esophageal atresia in a newborn with complex congenital heart disease. J Pediatr Surg 2007; 42: 1616–9. 17848260 10.1016/j.jpedsurg.2007.05.013

[11] Sampat K, Losty PD. Diagnostic and management strategies for congenital H-type tracheoesophageal fistula: a systematic review. Pediatr Surg Int 2021; 37: 539–47. 33474597 10.1007/s00383-020-04853-3 PMC8026411

[12] Durakbasa CU. Management of congenital isolated H-type tracheoesophageal fistula. Curr Chall Thorac Surg 2022; 4: 23.

[13] Karnak İ, Şenocak ME, Hiçsönmez A, et al. The diagnosis and treatment of H-type tracheoesophageal fistula. J Pediatr Surg 1997; 32: 1670–4. 9433996 10.1016/s0022-3468(97)90503-0

[14] Dai J, Pan Z, Wang Q, et al. Experience of diagnosis and treatment of 31 H-type tracheoesophageal fistula in a single clinical center. Pediatr Surg Int 2018; 34: 715–9. 29855692 10.1007/s00383-018-4293-6

[15] Garcia NM, Thompson JW, Shaul DB. Definitive localization of isolated tracheoesophageal fistula using bronchoscopy and esophagoscopy for guide wire placement. J Pediatr Surg 1998; 33: 1645–7. 9856885 10.1016/s0022-3468(98)90599-1

[16] Hua K, Liao J, Sun D, et al. The intraoperative localization in recurrent tracheoesophageal fistula after esophageal atresia repair: a comparative study. BMC Pediatr 2024; 24: 796. 39633340 10.1186/s12887-024-05293-x PMC11616127

[17] Dralle H, Sekulla C, Haerting J, et al. Risk factors of paralysis and functional outcome after recurrent laryngeal nerve monitoring in thyroid surgery. Surgery 2004; 136: 1310–22. 15657592 10.1016/j.surg.2004.07.018

[18] Merchavy S, Kassem K, Awawde R, et al. Intraoperative nerve monitoring parameters and risk of recurrent laryngeal nerve injury in thyroidectomy: a systematic review and meta-analysis. Biomedicines 2025; 13: 2516. 41153796 10.3390/biomedicines13102516 PMC12561866

[19] Taniyama Y, Okamoto H, Sato C, et al. Prevention and management of recurrent laryngeal nerve palsy in minimally invasive esophagectomy: current status and future perspectives. J Clin Med 2024; 13: 7611. 39768533 10.3390/jcm13247611 PMC11678675

[20] Chakedis J, Shirley LA, Terando AM, et al. Identification of the thoracic duct using indocyanine green during cervical lymphadenectomy. Ann Surg Oncol 2018; 25: 3711–7. 30076554 10.1245/s10434-018-6690-4 PMC6181776

[21] Jin S, Sugitani I. Narrative review of management of thyroid surgery complications. Gland Surg 2021; 10: 1135–46. 33842257 10.21037/gs-20-859 PMC8033047

